# Targeting of chimeric antigen receptor T cell metabolism to improve therapeutic outcomes

**DOI:** 10.3389/fimmu.2023.1121565

**Published:** 2023-03-14

**Authors:** Priyanka Maridhi Nanjireddy, Scott H. Olejniczak, Nataliya Prokopenko Buxbaum

**Affiliations:** ^1^ Department of Pediatric Oncology, Pediatric Oncology, Roswell Park Comprehensive Cancer Center, Buffalo, NY, United States; ^2^ Immunology Department, Roswell Park Comprehensive Cancer Center, Buffalo, NY, United States; ^3^ Department of Pediatrics, Roswell Park Comprehensive Cancer Center, Buffalo, NY, United States

**Keywords:** CAR T cell, adoptive cell therapy (ACT), cell metabolism, immunometabolism, tumor microenvironment

## Abstract

Genetically engineered chimeric antigen receptor (CAR) T cells can cure patients with cancers that are refractory to standard therapeutic approaches. To date, adoptive cell therapies have been less effective against solid tumors, largely due to impaired homing and function of immune cells within the immunosuppressive tumor microenvironment (TME). Cellular metabolism plays a key role in T cell function and survival and is amenable to manipulation. This manuscript provides an overview of known aspects of CAR T metabolism and describes potential approaches to manipulate metabolic features of CAR T to yield better anti-tumor responses. Distinct T cell phenotypes that are linked to cellular metabolism profiles are associated with improved anti-tumor responses. Several steps within the CAR T manufacture process are amenable to interventions that can generate and maintain favorable intracellular metabolism phenotypes. For example, co-stimulatory signaling is executed through metabolic rewiring. Use of metabolic regulators during CAR T expansion or systemically in the patient following adoptive transfer are described as potential approaches to generate and maintain metabolic states that can confer improved *in vivo* T cell function and persistence. Cytokine and nutrient selection during the expansion process can be tailored to yield CAR T products with more favorable metabolic features. In summary, improved understanding of CAR T cellular metabolism and its manipulations have the potential to guide the development of more effective adoptive cell therapies.

## Introduction

Surgery, chemotherapy, and radiation remain the cornerstones of cancer treatment. However, many patients are not cured by these approaches and those that are cured may incur significant morbidities, demonstrating an urgent need for the development of novel therapeutic options. Cancer cells subvert normal metabolic pathways to favor their growth and evasion of the immune system. Altered metabolism of the tumor microenvironment (TME) plays a critical role in tumorigenesis by creating an immunosuppressive milieu (1–4). Several therapeutic approaches to modify and restore the immune system are currently being developed and applied. Genetically engineered T cells constitute a powerful new therapeutic approach in the treatment of cancer. Chimeric Antigen Receptors (CARs) are synthetic receptors that graft a defined specificity onto an immune effector cell, typically a T cell, and augment T cell function. Once infused into the patient they expand and kill tumor cells. They also prevent tumor recurrence by promoting immune surveillance in conjunction with tumor infiltrating lymphocytes or by their own persistence (5–7). While significant strides have been made in CAR T cell therapy for hematologic malignancies leading to FDA approval of multiple products, durable responses in solid tumors remain limited (8, 9). Strategies to improve CAR T cell function are actively being sought. Metabolic manipulation represents a potential approach for improving the former given that T cell function is closely tied to cellular metabolism. Energetic demands and consequences of T cell activation, cytokine production, proliferation, and survival are facilitated by metabolic rewiring (10, 11). Furthermore, each stage within the CAR T manufacture process can influence the eventual metabolic profile of the infusion product, while metabolic features of the latter are directly linked to *in vivo* efficacy and persistence (8). The goal of this manuscript is to review known aspects of T cell metabolism, in the context of CAR T therapy, and present potential metabolic interventions that can be undertaken at each step of the manufacture process, including CAR design, priming, and expansion to leverage metabolic fitness of CAR T cells to augment therapeutic outcomes. In general, CAR T products containing memory like cells with enhanced mitochondrial fitness and high reliance on oxidative phosphorylation (OXPHOS) along with fatty acid oxidation (FAO) have been shown to have superior *in vivo* anti-tumor efficacy and long-term persistence. While effector function is tied to glycolysis that is engaged upon antigen-driven activation, a high proportion of memory cells that can be maintained in diminishing levels of antigen are associated with sustained anti-tumor responses. Thus, strategies that favor mitochondrial biogenesis along the manufacture process of the CAR T cells and maintenance of this metabolic phenotype after infusion are discussed in this review.

## Brief overview of T cell metabolism

**Figure 1:**
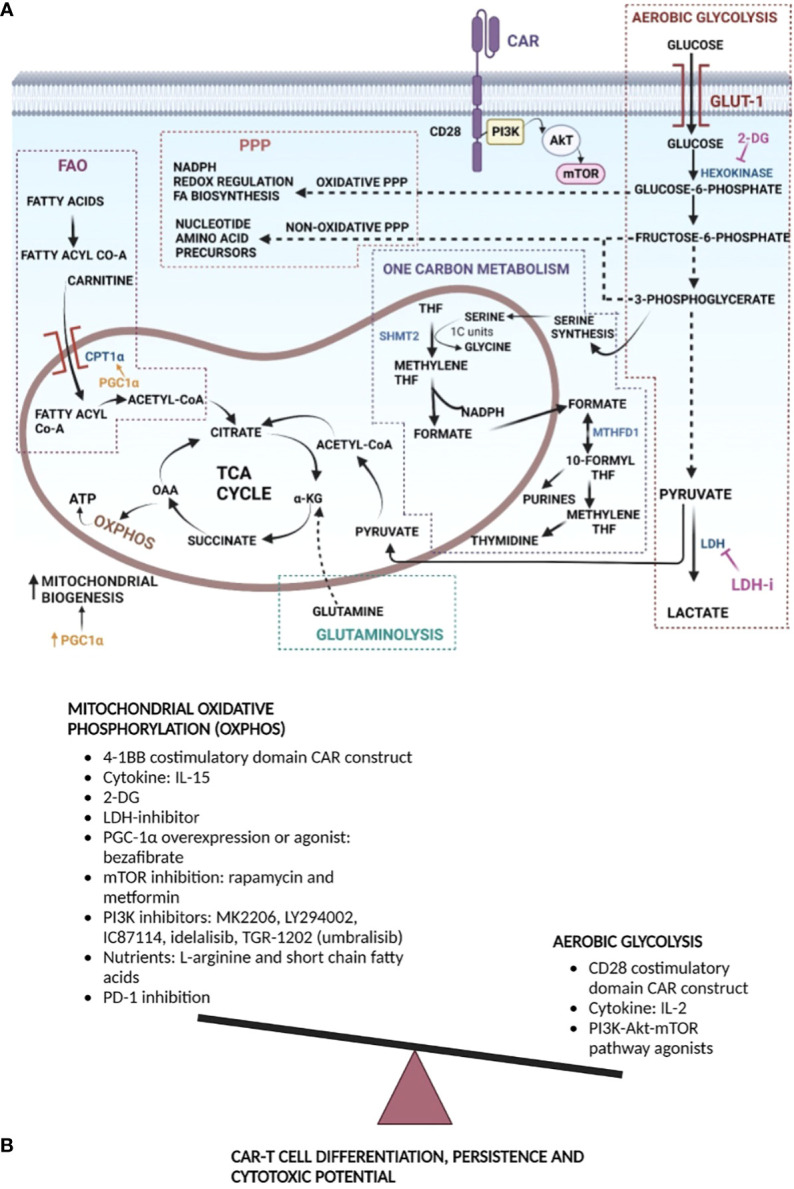
Metabolism manipulation strategies to improve CAR T cell efficacy and persistence: **(A)** T cells undergo metabolic rewiring upon encountering antigen. Aerobic glycolysis is upregulated upon T cell activation. Glycolysis provides energy for cell effector functions, as well as intermediates for the PPP that generates NADPH for anabolic processes and fuels nucleotide and amino acid biosynthesis. Proliferating T cells also rely on serine-glycine, folate, and methionine metabolism to generate one carbon units for *de novo* nucleotide synthesis, as well as NADPH production. Pyruvate produced at the end aerobic glycolysis can enter mitochondria for conversion to Acetyl CoA that feeds into the TCA cycle. Jointly, within the mitochondria, the TCA cycle, fatty acid oxidation, and glutaminolysis fuel T cell proliferation and differentiation. T cell activation is facilitated by increased mitochondrial biogenesis, generation of energy, OXPHOS and generation of reactive oxygen species, with NADPH providing reducing power for the latter. Potential metabolic points of stimulation or inhibition for enhancing CAR T cell function are provided. 2-DG inhibits hexokinase, re-directing metabolites toward PPP and OXPHOS. Inhibition of LDH drives pyruvate toward TCA and OXPHOS. PGC1α stimulates mitochondrial biogenesis, which promotes OXPHOS, and can stimulate FAO *via* CPT1α. **(B)** Generally, a memory like CAR T phenotype has been shown to improve *in vivo* persistence and anti-tumor function. Memory cells primarily rely on OXPHOS; hence, strategies that favor the former during CAR design, CAR T manufacture, expansion, or following infusion are provided. Mitochondrial OXPHOS can be increased directly or indirectly by decreasing aerobic glycolysis. Acronyms used: GLUT-1, glucose transporter-1; 2-DG, 2-deoxyglycose; LDH, lactate dehydrogenase; LDHi, lactate dehydrogenase inhibitor; α-KG, α-ketoglutarate; OAA- oxaloacetic acid; TCA Cycle, tricarboxylic acid cycle; THF, tetrahydrofolate; 1C, one carbon; SHMT2, serine hydroxymethyl transferase 2; MTHFD1, methylene tetrahydrofolate dehydrogenase 1; PPP, pentose phosphate pathway; FA, fatty acid; CPT1α, carnitine palmitoyl transferase 1α; PGC1α, peroxisome proliferator-activated receptor-gamma co-activator 1α; NADPH, reduced nicotinamide adenine dinucleotide phosphate; 4-1BB, distinct costimulatory molecule; CAR, chimeric antigen receptor; CD, cluster of differentiation; IL, interleukin; mTOR, mammalian target of rapamycin; PI3K, phosphatidylinositol 3-kinase.

T cells are specific effectors of the adaptive immune system, which continuously survey and eliminate pathogen infected cells as well as tumors. To elicit a robust immune response, T cells differentiate into diverse functional subsets, such as effector T cells (Teff) and memory T cells (Tmem), which can further differentiate into more diverse subsets based on cytokine milieu (12, 13). Such subsets have different functional and metabolic requirements. In the absence of antigen, naïve T cells are quiescent, rarely divide, have a low energetic demand, and continuously circulate through secondary lymphoid tissues (11, 14). Memory T cells are also mostly quiescent but display a greater mitochondrial mass that provides a bioenergetic advantage to support rapid recall responses upon antigen re-exposure. Both sets of T cells rely almost completely on the energy derived from mitochondrial oxidative phosphorylation (OXPHOS) and fatty acid oxidation (FAO) to maintain their basal energy level, cellular function, and viability (15–18). Following antigen exposure, T cell activation is orchestrated by TCR/peptide-MHC interaction providing the first signal forming an immune synapse. Further interaction at the synapse with costimulatory molecules provides the required second signal. Thus, a complete TCR-based activation of T cells requires two signals (15, 19, 20). Once activated, T cells predominantly engage in aerobic glycolysis, the pentose phosphate pathway (PPP), one-carbon metabolism, fatty acid oxidation (FAO), and glutaminolysis to facilitate proliferation and enable subsequent effector functions (17, 21–24), **Figure 1A**.

### Glycolysis

Naïve T cells and non-proliferating cells generate ATP *via* OXPHOS. Once T cells are activated, they engage glycolysis, where pyruvate is fermented to lactate in the cytoplasm in the presence of sufficient oxygen, a process termed the Warburg effect (24–27). The process of glycolysis begins with the uptake of extracellular glucose, mediated by cell surface transporter Glut1 and ends in conversion to pyruvate, through a series of enzymatic reactions generating metabolites that can enter other pathways (21, 28, 29). Pyruvate produced under aerobic conditions can be converted in the mitochondria into acetyl Co-A, which then enters the tricarboxylic acid (TCA) cycle. Alternatively, under anaerobic conditions, it can be converted in the cytoplasm into lactate, which is then excreted from the cell (30), **Figure 1A**. While T cells require mitochondrial ATP from OXPHOS for activation, continued proliferation of the activated T cells relies on either aerobic glycolysis or OXPHOS (29). Chang et al. showed that OXPHOS and aerobic glycolysis can be used interchangeably as fuel for T cell proliferation and survival, but glycolysis is essential for T cell effector function (29). Glycolysis while relatively inefficient for energy production compared to OXPHOS, which produces 10 times higher energy yield, may nonetheless be preferred by rapidly proliferating cells secondary to the concurrent generation of biosynthetic precursor molecules that the cell needs (18, 24, 31–34). Following T cell activation there is also a reduction of ATP output from the mitochondria, which is instrumental to maintaining a low ATP : ADP ratio, promoting a high glycolytic rate (18, 21, 32, 35). Glycolysis is critical for effector differentiation as it is required for the post-transcriptional regulation of interferon (IFN)-γ production (29). However, persistently heightened glycolysis limits the Teff capacity to establish immunological memory making them short lived, while moderately dampened glycolysis supports generation of long-lived memory CD8+ T cells (36). T cells activated in limited concentrations of glucose fail to upregulate cytotoxic molecules, such as perforin and granzyme (16). The PI3K-AKT pathway also regulates glycolysis. AKT activity augments glycolysis by inducing Glut1 trafficking to the cell surface, increasing the activity of key glycolytic enzymes and more importantly, activating the kinase, mammalian target of rapamycin (mTOR), that favors cell growth, protein synthesis and proliferation (16, 21, 31–33).

### Pentose phosphate pathway

Glycolysis is not the sole metabolic fate of glucose. The pentose phosphate pathway (PPP) starts from glucose-6-phosphate, an intermediate product of glycolysis, **Figure 1A**, and diverts it through several paths (37). The non-oxidative PPP branch shunts intermediates of glycolysis towards production of nucleotide and amino acid precursors that are needed for T cell growth and proliferation. Meanwhile, the oxidative PPP branch generates NADPH that is then used to generate reactive oxygen species (ROS) required for modulating redox balance and fatty acid biosynthesis (17, 37, 38).

### Glutaminolysis

Glutaminolysis is a pathway of incomplete glutamine oxidation that occurs in immune cells (39). The rate of glutamine utilization is high in both resting and activated lymphocytes. Glutaminolysis is critical for T cell function and as an energy producing pathway. Glutamine is a major anaplerotic fuel required for maintaining the TCA cycle (40) and reductive carboxylation in effector T cells (41, 42). Glutamine can enter the TCA cycle *via* conversion to α-ketoglutarate, **Figure 1A**, which can be processed to oxaloacetate, and subsequently citrate (40, 43). The latter is then excreted into the cytosol where it can be converted to acetyl CoA, the backbone of lipid synthesis. Glutaminolysis also provides metabolites for other biosynthetic processes by increasing availability of intracellular glutamine, aspartate, and ammonia that are necessary for purine and pyrimidine synthesis (44). A catabolic pathway involving glutamine, in the presence of NADP^+^ dependent malate dehydrogenase, following a series of steps, yields pyruvate and large amounts of NADPH. NADPH is required for protein, DNA, and RNA synthesis (44, 45). In contrast to glycolytic energy production, glutaminolytic energy production requires mitochondrial OXPHOS (34, 39, 46).

### One-carbon metabolism

While early studies in immune cells demonstrated glycolysis to be the predominant metabolic pathway (43, 47), more recent studies have shown a role for mitochondria in metabolic reprograming, respiration, and amino acid metabolism to support cellular proliferation (48, 49). Analysis of the mitochondrial proteome demonstrated that T cell activation induces mitochondrial proliferation and proteome remodeling, which then generates specialized mitochondria. The most striking change noted in these activated T cell mitochondria was the massive induction of enzymes involved in folate-mediated one carbon metabolism (50). One carbon metabolism is a key metabolic node in proliferating cells (51). It consists of serine-glycine metabolism, folate cycle and methionine cycle and is essential to processes such as *de novo* purine synthesis, methyl donor generation, as well as NADPH production that are critical for cell survival and function (52–54). Folate intermediates, such as, tetrahydrofolate (THF) are active carriers of one carbon units for *de novo* nucleotide synthesis (55). Ron-Harel et al. provided evidence for the former by showing a significant increase in the intracellular levels of precursors for these pathways during T cell activation, as well as ^13^C_2_-serine labeling of the media used to stimulate naïve T cells with tracing through the pathway to the yield of ^13^C labeled purines. This confirmed that metabolic changes in one carbon metabolism occur upon T cell activation and that T cells, upon activation, engage in both *de novo* biosynthesis and purine salvage pathways (50).

Serine is a major donor of one-carbon units during T cell activation and is essential for T cell effector responses (51). Other sources of one-carbon moieties for cytoplasmic one-carbon metabolism include formate, histidine, and purines (54, 56, 57). One-carbon units are generated in parallel pathways in the mitochondria and cytoplasm (50, 52, 54, 56, 57), and both sets of enzymes were found to be highly induced following T cell activation (50). However, the majority of one carbon units are generated within mitochondria in activated T cells. The key enzyme for mitochondrial one carbon metabolism and T cell survival is serine hydroxyl-methyltransferase-2 (SHMT2), **Figure 1A**. Evidence for the latter was generated in the SHMT2 knockdown (KD) model where a decrease in the one carbon unit pool, as well as an accumulation of metabolites upstream the *de novo* purine synthesis was observed (50). T cells from the SHMT2 KD displayed a 2- to 3-fold increase in cell death compared to wildtype. The latter was a result of increased cell death due a nucleotide imbalance, with an observed 50% reduction in purine levels but unaffected levels of pyrimidines leading to the inhibition of *de novo* purine synthesis and increased DNA damage. In addition, SHMT2 plays a critical role in glutathione synthesis; hence, the shortened T cell half-life was also attributable to increased oxidative stress promoting cell death under hypoxia in SHMT2 KD T cells (50). SHMT2 KD T cells could be rescued completely with the combination of formate, a product of mitochondrial one carbon metabolism, and N-acetyl cysteine (NAC), a glutathione precursor, reaffirming the important role of mitochondrial one-carbon metabolism in promoting T cell survival (50, 58, 59). Another critical regulator of CD4+ T cell proliferation and differentiation is methylenetetrahydrofolate dehydrogenase 2 (MTHFD2). Suguira et al. showed that MTHFD2 is selectively required for Teff cells. A deficiency of MTHFD2 alters *de novo* purine synthesis, resulting in insufficient generation of nucleotides (60). To summarize, one-carbon metabolism plays a major role in T cell proliferation and survival.

### Fatty acid oxidation

Fatty acid oxidation (FAO) is a pathway that converts fatty acids to acetyl-CoA, NADH, FADH2 which are then used by cells for energy production (38). This pathway takes place in the mitochondria and can produce tremendous amounts of ATP. Starting in the cytoplasm with activation of fatty acids to a fatty acid acyl-CoA, short chain fatty acids diffuse passively into the mitochondria, while medium and long chain fatty acids are conjugated to carnitine and are consequently shuttled into the mitochondria. Once inside the mitochondria, carnitine conjugated fatty acids are converted back to fatty acid acyl-CoA that undergoes β-oxidation generating large amounts of acetyl-CoA, NADH and FADH2, which are used in the TCA cycle and electron transport chain (ETC) to produce ATP (38).

When in circulation, naïve T cells engage in FAO for ATP production or use OXPHOS to maintain low levels of glycolysis. Once activated, they switch from FAO to fatty acid synthesis (FAS) due an increased demand for lipids (11, 35). FAO promotes memory T cell production that is necessary for a long-lived immune response. CD8+ memory T cells are dependent on FAO for their development, persistence, and immediate response to stimulation (20, 23, 61). This is enabled by their greater mitochondrial mass and spare respiratory capacity compared to naïve and effector counterparts (20, 61). Therefore, lipid metabolism plays an important role in T cell activation and formation of memory phenotypes.

## Brief overview of CAR T cell manufacture process and the associated cellular metabolism consequences

CARs are synthetic receptors that redirect T cells against a defined target in a major histocompatibility complex (MHC)-independent fashion. CAR T cell therapy aims to eliminate specific tumor cells in a sustained manner. The first step of the CAR T manufacturing process is the collection of PBMCs through leukapheresis, either patient’s own or from a donor (62, 63), followed by isolation of T cells. T cells are then activated with anti-CD3/CD28 magnetic beads to promote proliferation and differentiation. At this point, T cells transition from a naïve or quiescent state to an activated state, with a concurrent metabolic switch from FAO to glycolysis (20, 26, 29), whereby T cells differentiate into either high glucose requiring Teff cells and low glucose requiring Tmem cells (11, 36). The next step in the manufacturing process is viral transduction, where T cells are incubated with a lentiviral vector encoding the CAR construct. The final step in manufacture includes expansion within cytokine enriched media and represents yet another opportunity for T cells to differentiate into distinct functional phenotypes, i.e., Teff, Tem, Tscm, Tcm, based on culture conditions. CAR T products containing a high proportion Tcm or Tscm subsets have been shown to have an enhanced subsequent metabolic adaptability, mediated mainly through mitochondrial metabolism, and are able to maintain a long-term anti-tumor response *in vivo* (8, 10, 14, 64–67). Optimization of CAR T cell metabolism for the maintenance of early memory phenotypes, Tscm and Tcm, to improve CAR persistence and cytotoxic function can potentially be carried out at several stages, as described hereafter, **Figures 1A, B**.

CAR constructs: The first T cell activating receptors were CD3ζ chain fusions, which also elucidated the role of the ζ chain (68, 69). These early studies showed that T cell activation signaling, and initiation of cytotoxicity were possible by cross-linking the fusion receptors. Eshhar et al. then incorporated the immunoglobulin-derived single chain variable fragment (scFv) onto these receptors to direct and lyse hapten coated cells (5, 70), generating the first-generation CAR T. Since then, multiple generations of CAR T cells have been engineered. The current FDA approved CAR T cell receptor has four parts: 1) an extracellular target antigen-binding domain composed of a single chain variable fragment (scFv) of an antibody targeting the specific tumor antigen; 2) a hinge region; 3) a transmembrane domain; and 4) the intracellular domain, i.e., T cell receptor (TCR) signaling domain comprised of CD3ζ and costimulatory domains. The CD3ζ domain in the CAR structure serves as signal 1 and a costimulatory domain provides signal 2 (15). Second and third generation CARs have multiple costimulatory domains, such as immunoglobulin (Ig) superfamily members, CD28 (B7.1/B7.2-CD28) and inducible T cell costimulatory (ICOS, B7RP-1-ICOS) (71), and tumor necrosis factor receptor (TNFR) superfamily members 4-1BB, OX40 and CD27 (10, 15). Depending on the costimulatory domains incorporated into the CAR construct, different downstream signaling pathways are activated that impact *in vivo* persistence, susceptibility to exhaustion, generation of memory, and anti-tumor potency (10). CD28 and 4-1BB signaling domains are the most widely used and studied. Hence, their effects on CAR T cell metabolism are described below.

Metabolic phenotypes linked to CAR constructs: Kawalekar et al. found that 4-1BB based (BBζ) CAR T cells had higher proliferative capacity and persistence than CD28 based (28ζ) CAR T cells. Both sets of CARs started with a uniformly increased expression of CD69, an activation marker, on Day 1. Subsequently, the BBζ CAR T cells proliferated and persisted in culture for over 4 weeks, while the 28ζ CAR T cells had done so for 2 weeks (72). Even more striking persistence differences were observed *in vivo*, where CD28-based CAR T cells were detected for 30 days (73–75), while 4-1BB based CAR T cells persisted for years (76, 77). The increased persistence of BBζ CAR T cells was attributed to their differentiation into CD45RO^+^CCR7^+^ Tcm cells, and this phenotype was maintained through the culture process. In contrast, 28ζ T cell expansion resulted in a higher proportion of CD45RO^+^CCR7^-^ Tem cells following stimulation through the CAR (20, 72, 78).

To characterize cellular metabolism changes upon CAR signaling, Kawalekar et al. measured the oxygen consumption rate (OCR), a surrogate measure of OXPHOS, before activation, as well as at 7- and 21-days post antigen stimulation; including following serial addition of an inhibitor of ATP synthesis, an uncoupling ionophore, and blocking agents of the ETC. The OCR profiles were similar in both groups on Day 0. On days 7- and 21-post antigenic stimulation an ~10-fold increase in OCR was observed in both groups. Maximal respiratory capacity of BBζ CAR T cells showed a robust increase following decoupling of the mitochondrial membrane. The extracellular acidification rate (ECAR), a surrogate of lactic acid production during glycolysis, was elevated in 28ζ CAR T cells. 28ζ CARs were found to be rapidly consuming glucose and generating lactate, consistent with observed high ECAR levels. Additionally, Glut1, PDK1 (26), G6PD, and phosphoglycerate kinase (PGK), and have also been shown to be elevated in 28ζ cells. These findings support the conclusion that 28ζ CAR T cells rely on glycolysis, **Figure 1B**, for their energy needs (26, 72), a characteristic of Teff cells (72, 78).

In contrast, BBζ CAR T cells rely on mitochondrial oxidative phosphorylation (72). When glucose uptake and fatty acid utilization rates were evaluated by measuring residual glucose, lactate, and heavy-carbon-labeled long chain fatty acid, palmitic acid, in the media at different time points, BBζ cells showed high utilization of palmitic acid, measured by heavy-carbon-labeled acetyl CoA levels. As β-oxidation of fatty acids generates acetyl CoA, an increase in heavy-carbon-labeled acetyl-Co-A pool indicated that BBζ CAR T cells use FAO to fuel their bioenergetic needs. Also, carnitine palmitoyl transferase (CPT1A), which controls a rate limiting step in mitochondrial FAO and promotes mitochondrial biogenesis (20), was significantly elevated in BBζ cells. Additionally, fatty acid binding protein (FABP5) that is involved in fatty acid uptake, transport, and metabolism was also elevated in these cells. Together, these findings indicate that BBζ CAR T cells use fatty acids for their energy needs (23, 72). Finally, BBζ CAR T cells have a survival advantage due to their ability to generate increased mitochondrial mass (61, 72). BBζ CAR T cells consistently demonstrated high spare respiratory capacity (SRC), which is a characteristic of natural CD8^+^ T cell memory and supports T cell function in the hostile tumor environment (20, 72, 79–81).

Selection of CAR costimulation systems based on associated metabolic re-wiring: As discussed, the costimulatory signals in the CAR constructs that are necessary for T cell activation, expansion, cytokine secretion, cytotoxic function, memory formation, and survival are mediated through metabolic reprogramming. Depending on the costimulatory domain incorporated into the construct, different signaling pathways are triggered upon antigen activation (82–84). As already discussed, the CD28 domain in CARs leads to the activation of PI3K/Akt pathway, **Figure 1A** and aerobic glycolysis as the predominant metabolic program. In contrast, T cells with CAR constructs comprising of 4-1BB domain activate the NF-κB, MAPK, and ERK pathways. These CAR T cells exhibit enhanced OXPHOS and SRC derived from fatty acid oxidation. Mitochondrial biogenesis and oxidative metabolism associated with Tcm phenotype are preferred in CAR T therapy given enhanced *in vivo* persistence and function (72), **Figure 1B**.

Another costimulatory domain, ICOS, has been shown to lead to higher PI3K/Akt pathway activation compared to CD28, and increased secretion of IL-21, IL-17, and INF-gamma (85). OX40, a member of the TNFR family which is upregulated with T cell activation *via* the OX40L has a broad effect on T cell activation, proliferation, differentiation, and survival. It stimulates glycolysis and OXPHOS *via* PI3K/Akt, MAPK and NF-κB pathways, and induces antiapoptotic genes, Bcl-1and Bcl-xl, to promote T cell expansion and survival, respectively (86–88). To summarize, selection of co-stimulatory domains has a significant impact on persistence and antitumor function of CAR T therapies.

Improving CAR T cell efficacy using CAR systems encoding cytokines and/or chemokines: Cytokine- and chemokine- encoding genes can be added into the CAR construct, which may lead to distinct cellular metabolism features. While the metabolic consequences of such modifications have not been fully explored, they have been implemented to improve CAR T entry into and function within the TME. While first-generation CARs had a single CD3ζ signaling domain they were ultimately not very effective (89–91). Second and third generation CAR T cells have incorporated one or two costimulatory signals, respectively. Genetic modifications to CARs for co-expression of cytokine (92–98), chemokine (99–101), or both factors (102–104) have been successfully used to enhance therapeutic efficacy CAR T cells.

Use of cytokines in expansion media to promote CAR T cell metabolic rewiring: Cytokine composition in culture media impacts efficacy of the CAR T cell product. Cytokines that have been broadly investigated to date are IL-2, IL-7, IL-15, and IL-21 (105). IL-2 is a T cell growth factor that promotes effector differentiation and glycolysis in CD8 T cells (22, 106, 107). When CAR T cells are expanded using IL-2, they differentiate to effector CD8+ cells *via* Akt-m TOR pathway. However, aryl hydrocarbon receptor activation was also observed under such expansion conditions, suggesting that IL2 signaling contributes to CAR T cell exhaustion (105, 108). IL-7 and IL-15 can cause differentiation to memory T cells (20, 109, 110). IL-7 can induce glycerol channel expression and triglyceride (TAG) synthesis that results in a product with a high CD8 memory proportion. It also promotes Glut-1 cell surface expression thereby increasing glucose uptake and promoting cell survival (111). The combination of IL-2 and IL-7 in the *ex vivo* culture media during CAR T cell expansion enhances glycolysis and differentiation of T cells towards the effector phenotype necessary for cytotoxicity (112). Another cytokine used for CAR T expansion is IL-15. It downregulates mTORC1 activity and expression of several glycolysis enzymes, thereby improving mitochondrial fitness and maintenance of the Tscm phenotype (105, 113). Consequently, recent trials have used CAR T cells expanded in IL-7 and IL-15 and have demonstrated superior anti-tumor efficacy. Notably, CAR T cells expanded in IL-2 can show phenotypic features similar to those expanded in IL-15, when mTORC1 inhibition is provided by concurrent exposure to rapamycin (24).

Optimizing nutrients within expansion media: Optimization of expansion media, aside from cytokine milieu, can also impact on T cell differentiation and subsequent function. For example, L-arginine is consumed rapidly in activated T cells; hence, when exogenous L-arginine was supplemented, a shift from glycolysis to OXPHOS was observed in cultured cells (34, 114). A potential explanation for this switch is that with increased L-arginine leads to an upregulation of the serine biosynthesis pathway, fueling the TCA cycle and enhancing OXPHOS (114, 115). The decrease in glycolysis combined with increased L-arginine levels potentiates the generation of the Tcm subset, promoting anti-tumor activity *in vivo* (29, 36, 114). Another feature of T cells cultured in excess L-arginine is prolongation of survival (114). Other nutrients in the media that could be altered are fatty acids, especially short chain fatty acids (SCFA), such as butyrate, propionate, and acetate (116), which diffuse passively into the mitochondria. Depending on the concentration, the SCFAs have been shown to favor a memory-like T cell phenotype (116–120).

Following infusion, CAR T have to traffic to tumor sites, penetrate the TME, and persist in the patient to generate sustained anti-tumor activity. Strategies aimed at improving trafficking and persistence of CAR T, including nanoparticle RNA vaccines as well as oncolytic viruses have been described, albeit so far in pre-clinical studies, with clinical testing currently ongoing for the latter. While these approaches have the potential to enhance CAR T *in vivo* performance, they are not metabolic in nature and are reviewed elsewhere (121).

## The impact of TME metabolism on endogenous and adaptively transferred T cells

Cancer involves abnormal cell growth, diminished apoptosis, and evasion of the normal host defenses that facilitate local invasion and potential distant metastasis (122). The high replication rate of tumor cells requires a continuous source of energy necessitating modification of normal metabolism. Cancer cells engage aerobic glycolysis to meet their metabolic requirements and ATP production (1, 123). Increased aerobic glycolysis by the tumor cells creates a glucose deprived TME, impairing effector T cell function within the endogenous anti-tumor immunity and adaptive cell therapy (1, 123). Tumor derived lactate accumulation *via* aerobic glycolysis leads to an acidified TME. This in turn impedes monocarboxylate transporter-1 mediated, gradient-dependent export of lactate from CD8+ T cells (1, 124, 125). Malignant cells within a solid tumor rapidly proliferate and organize, which can lead to poor vascularization that exacerbates TME hypoxia. In addition to the decreased oxygen delivery, TME hypoxia can be further enhanced by high tumor oxygen consumption. Hypoxia inhibits differentiation, proliferation, and cytokine production of cytotoxic T lymphocytes (CTL) and T helper (Th1) cells (10, 125, 126). Under hypoxic conditions, tumor cells respond with rapid induction of transcription factor hypoxia-inducible factor 1 alpha (HIF1α) and NFκB (1, 127). Both factors are involved in the regulation of genes implicated in inflammation and adaptation to hypoxia. These pathways control mitochondrial dynamics and mitophagy, promote TME acidification, and regulate the expression of cytokines and angiogenic factors (1, 10, 128–131). These transcription factors regulate the expression of a plethora of cytokines and angiogenic factors, including, IL-6, IL-10, and vascular endothelial growth factor (VEGF) (10, 127). These molecules then recruit cells with immunosuppressive function, including myeloid-derived suppressor cells (MDSCs), T regulatory (Treg) cells, innate lymphoid cells (ILCs), tumor associated macrophages (TAMs), and cancer associated fibroblasts (CAFs). This establishes an immunosuppressive TME and suppresses antigen processing and presentation (10, 132), leading to immune evasion. Furthermore, HIFα controls the expression of checkpoint molecule programmed death-ligand 1 (PD-L1) on the surface of cancer cells and PD-1 expression on T cells, which negatively impacts T cell survival and effector functions *via* the PD1/PD-L1 axis (10, 125). Additionally, hypoxia dampens activity of adenosine kinase and adenosine deaminase leading to adenosine accumulation in the TME that favors immunosuppressive cells and inhibits anti-tumor T cell function (125). As described, the TME plays an important role in augmenting endogenous T cell and CAR T cell function. Several excellent reviews on TME metabolism as a barrier to immunotherapy and metabolic strategies to manipulate the former have been recently published (3, 4, 133). The focus of this review is the manipulation of CAR T metabolism to improve *in vivo* function and persistence, which is described hereafter.

## Immunometabolic interventions to improve CAR T *in vivo* efficacy and persistence

Increased persistence and survival are characteristics of an effective CAR T anti-tumor response (134). Presence of specific T cell subsets, such as those with minimal differentiation and increased self-renewal capacity, can enhance CAR T cell anti-tumor efficacy (135). As already described, metabolism plays a major role in this process, with effector T cells depending more on glycolysis and memory T cells relying on mitochondrial oxidation and FAO for their bioenergetic needs (136). Pre-clinical studies have shown that an infusion of a high proportion of naïve T cells (Tn), Tscm, or Tcm leads to superior anti-tumor efficacy compared to products with higher Tem or Teff content (137, 138). However, during the *ex vivo* expansion process, a large proportion of Tem and Teff are generated due to a high rate of glycolysis induced during activation and expansion that drives T cells towards terminally differentiated phenotypes (36). The cytokine milieu during *ex vivo* expansion, antigen activation, and costimulation, contribute to this process, as previously described. Given that the functional phenotype, i.e., Tscm, and favorable metabolic features are linked, maintenance of this phenotype represents a promising strategy for generating effective adoptive T cell therapies, **Figure 1B**.

Inhibition of glycolysis: 2-deoxyglucose (2-DG) is a prototypical inhibitor of the glycolytic pathway that blocks hexokinase (139), **Figure 1A**. Fraietta et al. showed inhibition of glycolysis with 2-DG decreased T cell effector and promoted memory T cell generation (140). Additionally, the same group demonstrated that a glycolytic gene signature characterized CAR T cells obtained from patients with partial or no response to therapy. The former also displayed a higher uptake of a glucose analog than CAR T cells isolated from patients with complete responses (140). Thus, employing strategies to interfere with glycolysis is a potential solution to improve downstream *in vivo* CAR T cell efficacy. At high concentrations, however, 2-DG may interfere with T cell proliferation and cytolytic function. Cham et al. showed that 2-DG at 10-50 mM concentrations in culture interfered with T cell proliferation and almost completely abolished cytolytic ability of CD8^+^ T cells (123). Shi et al. showed that inhibition of glycolysis by 2-DG at a concentration of 1mM had minimal inhibitory effects on cell proliferation (139). Similarly, Sukumar et al. used 2 mM concentrations of 2-DG, which sufficiently inhibited glycolysis without interfering with cell proliferation and successfully induced OXPHOS. They also demonstrated that T cells primed in the presence of 2-DG accumulated at higher numbers in tumors (36). In conclusion, inducing a memory T cell phenotype by inhibiting glycolysis with relatively low levels of 2-DG during CAR T cell manufacture is a potential strategy to improve CAR T cell efficacy, **Figure 1B**.

Improving mitochondrial function: As already discussed, mitochondria play a key role in the regulation of T cell metabolism, biosynthesis, migration, cell fate and programmed cell death. Regulating mitochondrial OXPHOS is one approach to improve CD8^+^ T cell function. Lactate dehydrogenase (LDH) is an enzyme just distal to glycolysis that converts pyruvate into lactate. Hermans et al. demonstrated that a small molecule LDH inhibitor, at a concentration of 1 uM led to metabolic rewiring, blocking generation of lactate and promoting pyruvate entry into the TCA cycle, and ultimately enhancing OXPHOS (51), **Figure 1A**. The latter can inhibit terminal effector differentiation and exhaustion. The same group also demonstrated that LDH inhibition in combination with IL-21 exposure increased the formation of Tscm cells leading to an improved anti-tumor response and persistence. Interleukin-21 (IL-21) is a cytokine that uses the common cytokine receptor γ chain (γ_c_) as a receptor component (141). It primarily activates STAT3 (142), along with IL-7 and IL-15 expands CD8+ T cells (143). Thus, transient inhibition of LDH in combination with IL-21 supplementation during expansion phase generated a more effective cell therapy product (51). Another strategy for improving mitochondrial function is through the peroxisome proliferator-activated receptor-gamma coactivator (PGC)-1α that belongs to a family of transcription coactivators (144). PGC-1α overexpression in CD8 T cells has been found to boost mitochondrial biogenesis and memory phenotype, enhancing anti-tumor immunity (121, 145, 146), **Figure 1A**. Additionally, overexpression of PGC-1α in exhausted T cells improved their mitochondrial function, restoring functionality (121, 147). CD8^+^ T cells with PGC-1α overexpression secondary to exposure to bezafibrate, a PGC-1α agonist, in the presence of PD-1 blockade, upregulated mitochondrial OXPHOS and increased FAO, which enhanced their survival (148). Bezafibrate is a drug already in clinical use for hypercholesterolemia and could potentially be deployed in the setting of adoptive immunotherapy, following CAR T infusion.

An exhausted T cell (Tex) phenotype has been described extensively in the setting of chronic infection but has also been recognized in tumor-resident endogenous T cells and in the setting of adoptive T cell therapy (105, 149). Tex vulnerability is at least in part attributable to tonic TCR stimulation, which leads to metabolic rewiring and epigenetic changes that can enforce terminal exhaustion (105). CAR T cells are thought to be especially susceptible to this process given continuous antigenic stimulation that occurs during their *ex vivo* expansion, ahead of exposure to tumor antigen in the TME. Enhancing mitochondrial fitness and the linked Tscm phenotype during priming and expansion appear to counteract this susceptibility (105). Tex display inhibitory receptors, diminished effector ability, and decreased proliferative capacity. Metabolically, these cells are characterized to mitochondrial dysfunction and decreased glycolysis utilization (105, 149). Interestingly, Tex functionality can be restored with metabolic manipulation, as described above, through PGC-1α overexpression (121, 147), further supporting the notion that ability to maintain OXPHOS and FAO metabolism are necessary to avoid Tex phenotype. Additionally, PD-1 blockade in T cells has been shown to drive increased FAO, enhancing their survival and memory phenotype (121, 150). However, terminally exhausted T cells may only partially respond to PD-1 blockade, presumably due to fixed epigenic modifications (149). Acetate supplementation, which can restore acetyl-CoA required for histone acetylation, can improve chromatic accessibility and restore functionality in CD8 T cells (149, 151). Other epigenetic modifiers may lower susceptibility or restore functionality in Tex and are explored in greater depth in other reviews (105, 149).

Sustained activation of the PI3K-Akt-mTOR pathway by activation beads, IL-2, or tonic signaling drives T cells towards terminal differentiation and inefficient tumor killing (152–154). mTOR is a main regulator of CD8 T cell differentiation. Inhibition of mTORC1 by rapamycin or metformin (AMPK activator) has been shown to enhance OXPHOS by increasing FAO, and ultimately promoting the CD8+ T memory phenotype (154, 155). In the setting of CAR T, use of rapamycin during priming and expansion promoted the memory phenotype and increased FAO metabolism (121, 152, 156, 157). The addition of PI3K inhibitors, including, MK2206, LY294002, IC87114, idelalisib, and TGR-1202, during expansion has been shown to maintain CAR T cells in a less differentiated state leading to increased downstream anti-tumor efficacy and persistence of CAR T cells *in vivo* (105, 121, 158).

To summarize, induction of mitochondrial biogenesis and promotion of T cell differentiation towards a memory phenotype is a promising strategy for improving anti-tumor efficacy and persistence of CAR T cells.

## Conclusion

Aside from identifying tumor specific antigens and engineering appropriate CARs, several strategies to enhance *in vivo* CAR T function are currently being pursued. As greater understanding of T cell metabolism and immunosuppressive features of the TME is gained, this knowledge can be potentially leveraged to enhance anti-tumor responses. As described, manipulation of the T cell activation machinery that is linked to cellular metabolism reprogramming can enhance *in vivo* CAR T cell performance and persistence. Improved trafficking and function of CAR T cells in the TME have been observed with CAR T products containing distinct functional phenotypes that are linked to corresponding metabolic rewiring. These phenotypes can be generated *via* manipulations of the CAR construct itself, i.e., through selection of costimulatory elements, or during the expansion phase using cytokines and nutrients to skew CAR T products towards more favorable metabolic characteristics. Additionally, metabolic regulators can be used either during expansion or after infusion to generate and maintain metabolic phenotypes within CAR T cells, respectively. Metabolic rewiring of cellular therapies represents a promising clinically relevant approach to improve immunotherapy responses.

## Author contributions

All authors contributed to the writing of this review after NB received the invitation to submit.

## Glossary

**Table:**

2DG	2-deoxy-D-glucose
ADP	Adenosine diphosphate
Akt	Ak strain transforming
AMPK	AMP-activated protein kinase
ATP	Adenosine triphosphate
CAF	Cancer associated fibroblast
CAR-T	Chimeric antigen receptor T cell
CPT1A	Carnitine palmitoyl transferase 1A
CTL	Cytotoxic T lymphocyte
ECAR	Extracellular acidification rate
ERK	Extracellular signal-regulated kinase
ETC	Electron transport chain
FABP5	Fatty acid binding protein 5
FAO	Fatty acid oxidation
FAS	Fatty acid synthesis
G6PD	Glucose-6-phosphate dehydrogenase
HIF1α	Hypoxia inducible factor 1α
ICOS	Inducible T cell co-stimulator
IFN-γ	Interferon-γ
IL	Interleukin
ILC	Innate lymphoid cell
KD	knockdown
LDH	Lactate dehydrogenase
MAPK	Mitogen-activated protein kinase
MDSC	Myeloid-derived suppressor cell
MHC	Major histocompatibility complex
MTHFD2	Methylene tetrahydrofolate dehydrogenase-1
mTOR	mammalian target of rapamycin
NAC	N-acetyl cysteine
NADPH	Nicotinamide adenine dinucleotide phosphate
NF-κB	Nuclear factor kappa light-chain enhancer of activated B cells
OCR	Oxygen consumption rate
OXPHOS	mitochondrial oxidative phosphorylation
PBMC	Peripheral blood mononuclear cell
PD1	Programmed death-1
PDK1	Pyruvate dehydrogenase kinase-1
PDL1	Programmed death ligand-1
PGC1α	Peroxisome proliferator-activated receptor-gamma coactivator-1 alpha
PGK	Phosphoglycerate kinase
PI3K	Phosphatidylinositol 3-kinase
PPP	Pentose phosphate pathway
ROS	Reactive oxygen species
SCFA	Short chain fatty acid
scFv	single chain variable fragment
SHMT2	Serine hydroxy methyltransferase 2
SRC	Spare respiratory capacity
TAM	Tumor-associated macrophage
Tcm	central memory T cell
TCA	Tricarboxylic acid
TCR	T cell receptor Teff, effector T cell
Tem	effector memory T cell
Tex	exhausted T cell
Th1	T helper 1
Th17	T helper 17
THF	Tetrahydrofolate
Tmem	memory T cell
Tn	naïve T cell
TNFR	Tumor necrosis factor receptor
Treg	regulatory T cell
Tscm	stem cell-like memory T cell
VEGF	Vascular endothelial growth factor
